# PPARβ/δ priming enhances the anti-apoptotic and therapeutic properties of mesenchymal stromal cells in myocardial ischemia–reperfusion injury

**DOI:** 10.1186/s13287-022-02840-0

**Published:** 2022-04-23

**Authors:** Charlotte Sarre, Rafael Contreras-Lopez, Nitirut Nernpermpisooth, Christian Barrere, Sarah Bahraoui, Claudia Terraza, Gautier Tejedor, Anne Vincent, Patricia Luz-Crawford, Kantapich Kongpol, Sarawut Kumphune, Christophe Piot, Joel Nargeot, Christian Jorgensen, Farida Djouad, Stéphanie Barrere-Lemaire

**Affiliations:** 1 IGF, Université de Montpellier, CNRS, INSERM, 141 rue de la Cardonille, 34094 Montpellier Cedex 5, France; 2 IRMB, Univ Montpellier, INSERM, Montpellier, France; 3 IBRU, Department of Cardio-Thoracic Technology, Faculty of Allied Health Sciences, Naresuan University, Phitsanulok, Thailand; 4 Laboratorio de Inmunología Celular y Molecular, Facultad de Medicina, Universidad de los Andes, Santiago, Chile; 5 IMPACT, Center of Interventional Medicine for Precision and Advanced Cellular Therapy, Santiago, Chile; 6 School of Allied Health Sciences, Walailak University, Nakhon Si Thammarat, Thailand; 7 Département de Cardiologie Interventionnelle, Clinique du Millénaire, Montpellier, France; 8 CHU Montpellier, 34295 Montpellier, France

**Keywords:** Myocardial infarction, Reperfusion injury, Apoptosis, Mesenchymal stem cells, Priming, PPARβ/δ, Cardioprotection

## Abstract

**Background:**

Mesenchymal Stromal Cells (MSC) have been widely used for their therapeutic properties in many clinical applications including myocardial infarction. Despite promising preclinical results and evidences of safety and efficacy in phases I/ II, inconsistencies in phase III trials have been reported. In a previous study, we have shown using MSC derived from the bone marrow of PPARβ/δ (Peroxisome proliferator-activated receptors β/δ) knockout mice that the acute cardioprotective properties of MSC during the first hour of reperfusion are PPARβ/δ-dependent but not related to the anti-inflammatory effect of MSC. However, the role of the modulation of PPARβ/δ expression on MSC cardioprotective and anti-apoptotic properties has never been investigated.

**Objectives:**

The aim of this study was to investigate the role of PPARβ/δ modulation (inhibition or activation) in MSC therapeutic properties in vitro and ex vivo in an experimental model of myocardial infarction.

**Methods and results:**

Naïve MSC and MSC pharmacologically activated or inhibited for PPARβ/δ were challenged with H_2_O_2_. Through specific DNA fragmentation quantification and qRT-PCR experiments, we evidenced in vitro an increased resistance to oxidative stress in MSC pre-treated by the PPARβ/δ agonist GW0742 versus naïve MSC. In addition, PPARβ/δ-priming allowed to reveal the anti-apoptotic effect of MSC on cardiomyocytes and endothelial cells in vitro. When injected during reperfusion, in an ex vivo heart model of myocardial infarction, 3.75 × 10^5^ PPARβ/δ-primed MSC/heart provided the same cardioprotective efficiency than 7.5 × 10^5^ naïve MSC, identified as the optimal dose in our experimental model. This enhanced short-term cardioprotective effect was associated with an increase in both anti-apoptotic effects and the number of MSC detected in the left ventricular wall at 1 h of reperfusion. By contrast, PPARβ/δ inhibition in MSC before their administration in post-ischemic hearts during reperfusion decreased their cardioprotective effects.

**Conclusion:**

Altogether these results revealed that PPARβ/δ-primed MSC exhibit an increased resistance to oxidative stress and enhanced anti-apoptotic properties on cardiac cells in vitro*.* PPARβ/δ-priming appears as an innovative strategy to enhance the cardioprotective effects of MSC and to decrease the therapeutic injected doses. These results could be of major interest to improve MSC efficacy for the cardioprotection of injured myocardium in AMI patients.

**Supplementary Information:**

The online version contains supplementary material available at 10.1186/s13287-022-02840-0.

## Background

Myocardial reperfusion by revascularization of the culprit artery is the only treatment for AMI (Acute Myocardial Infarction) patients [1]. However, there is no treatment to specifically prevent irreversible ischemia–reperfusion (IR) injury, considered as the side effects of reperfusion mainly due to apoptosis and triggered by abrupt oxygen restoration in the ischemic tissue. Apoptosis is a highly regulated energy-dependent form of cell death of potential interest for therapeutic intervention because observed in ischemic disease both in animal models and in patients [2–5]. This regulated mechanism of cell death is specific to the reperfusion phase. Indeed, apoptosis is pre-activated during ischemia and executed during reperfusion, due to its dependence on ATP production [6]. Therefore, strategies to target specifically apoptotic cascades post-AMI need to be developed to prevent reperfusion injury and to limit infarct size [7–16].

Mesenchymal Stromal Cell (MSC)-based therapy has been reported to improve the post-MI functional recovery of the myocardium via a myriad of pathways that include the increase in endogenous cell survival, proliferation and angiogenesis. In addition, MSC potently repress inflammation and apoptosis through their plasticity and their capacity to release bioactive molecules and transfer organelles such as mitochondria and extracellular vesicles (EV) [17, 18]. Based on these properties, MSC have been tested in several preclinical studies with promising results. Indeed, in preclinical studies of AMI, MSC injection improves tissue repair and cardiac function [19], regulates the inflammatory response, decreases cell apoptosis [20] and reduces mortality in animals [21]. In clinical trials, although the safety and efficacy of MSC in phase I and II have been demonstrated, inconsistencies in phase III trials to evidence their cardioprotective properties have been reported [22–24]. These inconsistencies have been, in part, attributed to the poor in vivo survival rate and engraftment of MSC but also to the source of MSC used and the deleterious effects of the in vitro amplification process of MSC obtained from patients [24]. Therefore, to optimize MSC therapeutic potential and bring them into the routine clinical practice, the enhancement of MSC survival rate and engraftment in the damaged tissue as well as their anti-apoptotic properties on cardiac cells appears as a promising approach.

Cardiomyocytes are one of the main and most abundant cardiac cell types in the heart allowing contractile and pacemaker properties. Endothelial cells constitute the majority of non-cardiomyocytes and are likely to play a crucial role in physiological function and response to injury [25]. During IR injury, despite their higher tolerance to ischemic injury in close relation to their anaerobic metabolism, these two cell types undergo death through inflammation, oxidative stress and calcium overload [26, 27]. In particular, cardiac microvascular endothelial cell injury may occur much earlier and with much greater severity than cardiomyocyte injury [27]. Cardiomyocytes and endothelial cells seem to be particularly vulnerable during reperfusion, and undergo apoptosis within few minutes of reperfusion in isolated myocardial tissue [28, 29].

Approaches to improve MSC-based anti-apoptotic effects on cardiomyocytes have shown promising results both in vitro and in vivo [30–33] but only very few studies focused on endothelial cells [34]. However, these properties need to be further enhanced to optimally protect the heart from the massive apoptotic burst occurring during ischemia–reperfusion injury. Moreover, MSC do not survive long after transplantation into injured organs such as ischemic hearts. Indeed, within 24 h after systemic injection, only 3% of MSC are found in the marginal zones of the infarct myocardium, and less than 1% of MSC survive for more than a week [35]. Therefore, to significantly improve MSC-based therapy, the enhancement of both MSC anti-apoptotic properties and MSC survival abilities should be considered.

Peroxisome proliferator-activated receptors (PPARs) are nuclear receptors that exist in three different isoforms: PPARα, PPARβ/δ and PPARγ. They heterodimerize with RXR (retinoid X receptor) and, after ligand binding, act as transcriptional regulators. Dependent on the tissue ligands and cofactors, PPAR isoforms exert multiple functions [36]. While PPARα is mainly expressed in brown adipose tissue, intestine, heart, liver, kidney, PPARγ is expressed in immune cells, gut, white and brown adipose tissue. PPARβ/δ, ubiquitously expressed, is a proangiogenic member in vascular cell [37–39], where it plays an anti-apoptotic role through an elevation of cAMP [40]. Later studies showed that the anti-apoptotic properties of PPARβ/δ in endothelial cells were mediated through the upregulation of 14–3-3 mRNA and protein levels [41, 42]. In line with these studies, the selective PPARβ/δ agonist, GW501516, was shown to protect the cardiomyoblast cell line H9c2 from H_2_O_2_-induced cell death [43]. Although the anti-apoptotic effect of PPARβ/δ has been evidenced in cardiomyocytes, the modulation of PPARβ/δ expression, highly expressed by MSC [44], on MSC anti-apoptotic and cardioprotective properties has never been investigated.

The present study investigates the role of PPARβ/δ on MSC (i) survival and anti-apoptotic functions on cardiomyocytes and endothelial cells in vitro and (ii) therapeutic potential in an experimental model of MI.

## Methods

### MSC culture

MSC isolation and amplification of murine MSC were carried out in conditions previously described [45]. MSC were then seeded at a density of 0.5 × 10^6^ cells per cm^2^ in minimum essential medium (MEM)-α supplemented with 10% fetal bovine serum (FBS), 2 mM glutamine, 100 U/mL penicillin, 100 mg/mL streptomycin and 2 ng/ml human basic fibroblast growth factor (bFGF). The phenotypic and differentiation potential of MSC were assessed as previously described [45]. Briefly, for the phenotypic characterization, MSC were incubated during 20 min with conjugated monoclonal antibodies from BD Biosciences (Le Pont de Claix, France) in Phosphate Buffer Saline (PBS) supplemented with 0.1% bovine serum albumin. Regarding the differentiation of MSC into adipocytes, osteoblasts and chondrocytes, we relied on the inductive conditions already reported [45]. Adipogenic differentiation of MSC was assessed by the analysis of lipid droplets formation after Oil red O staining and by Reverse Transcriptase-quantitative Polymerase Chain Reaction (RT-qPCR). MSC chondrogenic differentiation was evaluated by RT-qPCR and the osteogenic differentiation was measured by RT-qPCR and the mineralization of the extracellular matrix. Induction of apoptosis was obtained by incubating MSC during 4 h in a minimal medium (without FBS) containing 350 μM of H_2_O_2_.

### MSC pre-treatments with PPARβ/δ agonist and antagonist and labeling with CM-DiI

MSC were pre-incubated for 24 h with either PPARβ/δ antagonist GSK0660 (0.1 and 1 μM) or PPARβ/δ agonist GW0742 (0.1 and 1 μM) before to be washed with PBS and used in co-cultures experiments or injected in the myocardium. When indicated, MSC were labeled with the fluorescent cell-tracker CM-DiI (Molecular Probes). MSC were collected and suspended in 5 mL PBS containing CM-DiI (10^7^ cells/10 μg) prior to be incubated at 37 °C for 5 min followed by 15 min at 4 °C, in the dark. Labeled MSC were washed 2 times in PBS, resuspended in Tyrode solution and maintained at 4 °C prior to be injected in the myocardium.

### Co-cultures of cardiomyocytes or endothelial cells with MSC

H9c2 embryonic rat heart-derived (ventricular) cells (myoblasts) were cultured in Dulbecco’s modified Eagle’s medium (DMEM), supplemented with 10% fetal bovine serum (FBS), 100 Units/mL penicillin, and 100 µg/mL streptomycin. To induce apoptosis, H9c2 were cultured in a minimal medium (without FBS) containing 350 μM of H_2_O_2_ during 4 h.

Human umbilical vein endothelial cells EA.hy926 (ATCC) were cultured in DMEM, supplemented with 10% fetal bovine serum (FBS), 100 Units/mL penicillin, and 100 µg/mL streptomycin. To induce apoptosis in EA.hy926, cells were cultured in a minimal medium (without FBS) containing 350 μM of H_2_O_2_ during 4 h.

In order to evaluate the effect of MSC on cardiomyocytes or endothelial cells, a co-culture assay was designed using the transwell system to avoid cell–cell contact with 50,000 MSC (upper part) in a complete medium containing FBS. After 1 h, H9c2 or EA.hy926 cells were evaluated for apoptosis using the DNA fragmentation assay.

### DNA fragmentation assay

Specific DNA fragmentation as a hallmark of apoptosis was quantified in vitro on cell lysates with an enzyme-linked immunosorbent assay kit (Cell Death ELISA; *Roche*) designed to measure histone-complexed DNA fragments (mono- and oligonucleosomes) released in the cytosol of cells induced for apoptosis. Data were normalized to the value by obtained in basal conditions of culture.

#### RT-qPCR

Isolation of total RNA for each sample was performed using the RNeasy Mini Kit (*Qiagen*). The quantity and quality of the RNA extracted were defined by using a NanoDrop ND-1000 spectrophotometer (NanoDrop ND, *Thermo Fisher Scientific*). Then, using the SensiFAST cDNA synthesis kit (*Bioline*), cDNA was synthesized by reverse transcribing 500 ng RNA into cDNA. Using the SensiFAST™ SYBR (*Bioline*) and a LightCycler® 480 Detection system Quantitative following manufacturer’s recommendations, PCR were performed. Primers specific were designed using the Primer3 software (sequences for primers are detailed in Additional file 1). Data were normalized to the housekeeping gene ribosomal protein S9 (RPS9) and the values were expressed as relative mRNA level of specific gene expression as calculated using the 2^−ΔCt^ method.

### Animal experiments

All experiments were carried out on C57BL/6 J mice (*Charles River laboratories*) in accordance with the European Communities Council directive of November 1986 and conformed to the "Guide for the Care and Use of Laboratory Animals" published by the US National Institutes of Health (NIH publication 8th Edition, 2011).

### *Langendorff *ex vivo* studies*

C57Bl6 male mice were anesthetized with a first intramuscular injection of an anesthetic cocktail comprising ketamine (14 mg/kg, Imalgène®; *Merial*) and xylazine (14 mg/kg, Rompun®; *Bayer*), and by a second injection of pentobarbital (76.6 mg/kg; *Sanofi-Aventis*). After sternotomy, the heart was excised, quickly cannulated through the aorta and mounted on a homemade Langendorff system. It was retrogradely perfused at pressure- and temperature-constant (37 °C) Tyrode solutions.

### Ischemia–reperfusion protocols

On the Langendorff system, the heart was subjected to ischemia–reperfusion (IR) protocol comprising, after a 15 min-stabilization period of Tyrode perfusion, a 30 min-period of no-flow to induce global ischemia. Reperfusion was achieved by restoring the perfusion during 60 min with the Tyrode solution alone (IR control) or with MSC cell in Tyrode solution (MSC) administrated during reperfusion. Ischemic postconditioning was induced at the end of global ischemia by the application of a conditioning stimulus comprising 3 cycles of 1 min-reperfusion / 1 min-ischemia, followed by 54 min of reperfusion. For the ex vivo evaluation of MSC-based cell therapy, MSC cells prepared in the Tyrode solution at various concentrations (2500; 5000 and 10,000 cells/mL) were perfused in isolated hearts during the 60 min-reperfusion phase (perfusion of ≈120 mL per heart).

### Infarct size assessment

LV were sliced transversally into 1-mm-thick sections and incubated in a 1% solution of 2,3,5-triphenyltetrazolium chloride (TTC, *Sigma-Aldrich*) for 15 min at 37 °C. After fixation in a 4% paraformaldehyde–PBS solution, the slices were weighted and each side was photographed (*Olympus* camera). Infarcted area was measured by planimetry with ImageJ software (*U. S. National Institutes of Health*) and expressed as a percentage of the left ventricle.

### Immunoblotting

Tissue samples (left ventricle) were rapidly frozen in liquid nitrogen after the end of ex vivo IR protocols. Samples were homogenized with a grinder in RIPA buffer [50 mM Tris (*Sigma-Aldrich*), 150 mM NaCl (*Sigma-Aldrich*), 1% Triton X-100 (*Sigma-Aldrich*), 0.1% sodium dodecyl sulfate (*Sigma-Aldrich*) pH 8.0] supplemented with EDTA-free protease and phosphatase inhibitor (Halt™ Protease and phosphatase single-use inhibitor Cocktail-100X, *Thermoscientific*). After centrifugation, pellets were resuspended in RIPA buffer for protein purification and incubated for 1 h at 4 °C. Protein concentrations were determined with the bicinchoninic acid (BCA) protein assay kit (*Pierce*). Samples (25 µg) of protein were resolved by SDS polyacrylamide gel electrophoresis (4–20% mini-Protean®TGX™ precast gels; *Biorad*) and transferred to nitrocellulose (Trans-Blot®Turbo™; *Biorad*). The following antibodies and suppliers were used: 1) *EMD Millipore*: anti-caspase 3 (Upstate 06–735), 2) *Cell Signaling Technology:* anti-phospho ERK1,2; anti-ERK1,2; anti-phospho AKT; anti-AKT; β-; anti-α-actinin; vinculin (E1E9V), 3) *Jackson ImmunoResearch:* horseradish peroxidase-conjugated anti-rabbit. Protein bands were visualized by enhanced chemiluminescence method using an ECL kit (SuperSignal™ West Pico chemiluminescent Substrate; *Thermo Scientific*™). Densitometry analysis was performed using Chemidoc™ (*Biorad*) and ImageJ software (*U. S. National Institutes of Health*). Signal intensities of each protein band were corrected with the corresponding value obtained for the loading controls (vinculin or α-actinin) and then normalized with the mean value obtained for the IR control conditions.

### Statistical analysis

Statistical analysis was performed only for n ≥ 5 independent experiments. Data (mean ± SD) were analyzed with ANOVA parametric test after confirmation of the normality of the population (Anderson–Darling Test for normal distribution). In case of non-conformity, nonparametric Kruskal–Wallis followed by Dunn’s post hoc test for multiple comparison or Mann–Whitney for two groups were used. Data were analyzed with GraphPad Prism (version 8.3.0 for MacOs, GraphPad Software, San Diego, California USA, www.graphpad.com). Statistical significance was noted as ns for *p* > 0.05, * for *p* < 0.05, ** for *p* < 0.01, *** for *p* < 0.001 and **** for *p* < 0.0001.

## Results

### Increased resistance of PPARβ/δ-primed MSC to H_2_O_2_-induced stress

Our previous study demonstrated in an ex vivo model of myocardial IR injury that MSC-induced cardioprotection was lost when PPARβ/δ expression in MSC was knocked out [46]. To determine the role of PPARβ/δ in MSC administered at reperfusion in an infarcted heart, we designed an in vitro protocol mimicking the stress received by MSC during transplantation and evaluated the properties of MSC pharmacologically activated or inhibited for PPARβ/δ.

First, we phenotypically and functionally characterized MSC used for the study as previously described [45]. We showed that MSC were negative for CD11b and CD45 and positive for markers expressed on MSC such as CD44 and Sca-1 (Additional file 1: Fig. 1A). After induction of MSC differentiation toward the three main mesenchymal lineages, MSC gave rise to: (1) chondrocytes as shown by the expression of *collagen type II* (*Col2*), (2) adipocytes characterized by the expression of *pparγ* and *lpl* and the formation of lipid droplets, and (3) osteoblasts characterized by the expression of *osteocalcin* (*OC*) and *alkaline phosphatase* (*ALP*) and Alizarin Red S staining (Additional file 1: Fig. 1B).

**Fig. 1:**
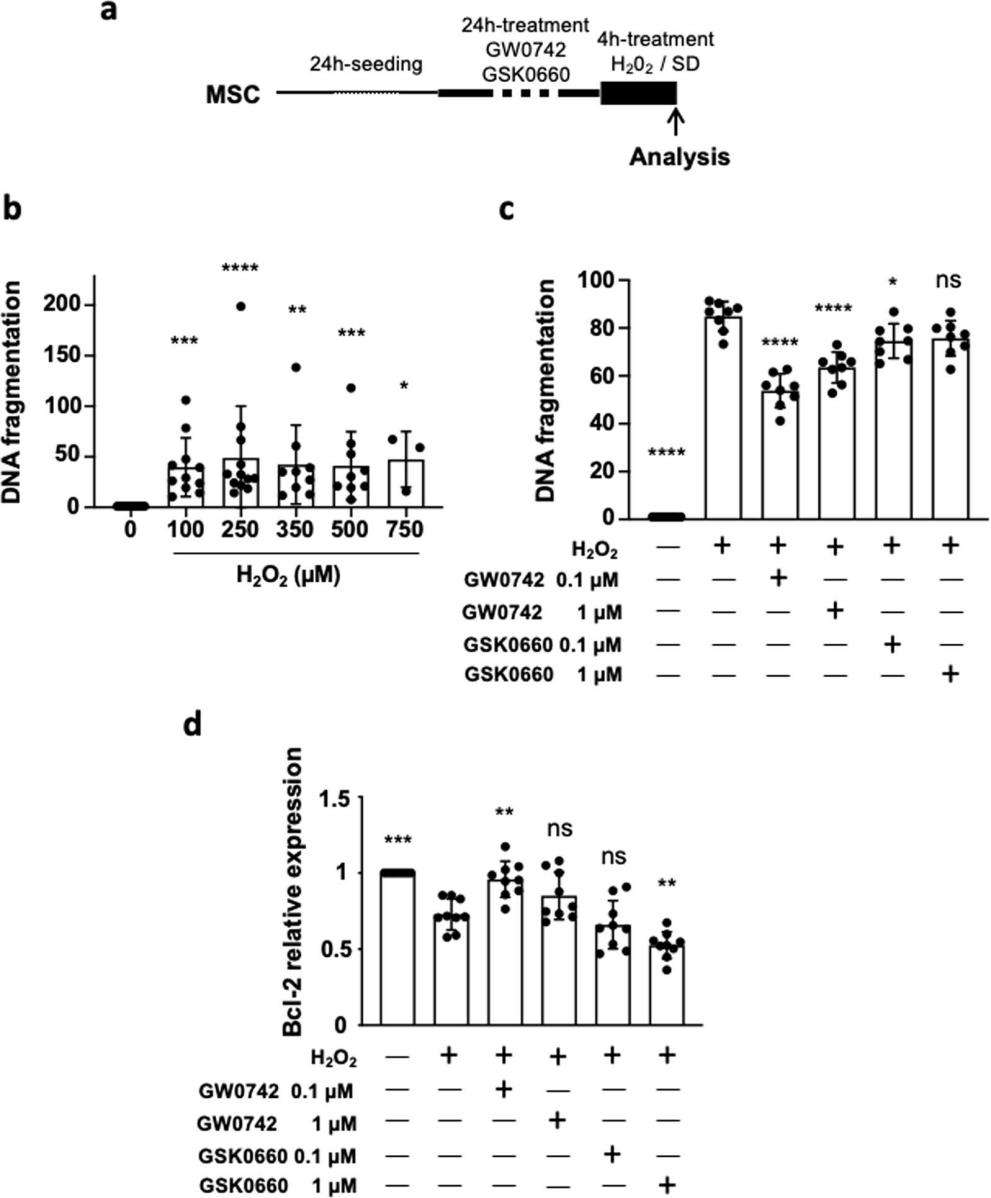
Decreased apoptosis in PPARβ/δ-primed MSC exposed to H_2_O_2_ stress. **a** 48 h after the seeding step in complete medium, MSC were subjected to H_2_O_2_-induced oxidative stress during 4 h in minimal medium with serum deprivation. Pharmacological preconditioning of MSC using PPARβ/δ agonist or antagonist was performed 24 h before oxidative stress exposure. At the end of the protocol, cell death or relative gene expression was analyzed measuring specific DNA fragmentation by ELISA and transcript amounts by RT-qPCR. **b** Scatter plot and bars were presented for specific DNA fragmentation quantification measured in MSC treated with H_2_O_2_ at various concentrations (0, 100, 150, 250, 500 and 750 µM) for 4 h in minimal medium. Data (normalized to the value obtained for MSC without H_2_O_2_) were plotted as scatter dot blots and mean ± SD for each group of treatment and compared using Kruskal–Wallis (Dunn’s post hoc test) and were noted vs the 0 µM H_2_O_2_ group (*n* = 15, independent cultures) ** for *p* = 0.0003 (100 group, *n* = 10), **** for *p* < 0.0001 (250 group, *n* = 12), ** for *p* = 0.0011 (350 group, *n* = 9), *** for *p* = 0.0010 (500 group, *n* = 9) and * for *p* = 0.0296 (750 group, *n* = 3). **c** Scatter plot and bars were presented for specific DNA fragmentation quantification measured in MSC pre-treated with GW0742 or GSK0660 at 0.1 and 1 µM 24 h before H_2_O_2_ exposure (250 µM, 4 h). Data (normalized to the value obtained for MSC without H_2_O_2_) were plotted as scatter dot blots and mean ± SD for each group of treatment and compared using ANOVA followed by the Tukey’s post hoc test (normality test passed). P values vs MSC/ H_2_O_2_ were noted **** for *p* < 0.0001 (MSC without H_2_O_2_), vs 0.1 µM GW0742 and vs 1 µM GW0742), * for *p* = 0.0402 (MSC/H_2_O_2_ vs 0.1 µM GSK0660) and ns for *p* = 0.0582 (MSC/H_2_O_2_ vs 1 µM GSK0660). In each case, *n* = 8 independent cultures were evaluated. **d** At the end of the protocol, mRNA expression levels in untreated or preconditioned MSC exposed to an H_2_O_2_ were assessed by RT-qPCR for *Bcl-2:* data (mean ± SD; *n* = 8 for each group) normalized to the control values were compared using ANOVA and the Tukey’s post hoc test (normality test passed). P values vs MSC/H_2_O_2_ were noted for *** for *p* = 0.0001 (MSC without H_2_0_2_), ** for *p* = 0.0015 (GW0742 0.1 µM), ns for *p* = 0.2379 (GW0742 1 µM), ns for *p* = 0.8168 (GSK0660 0.1 µM) and ** for *p* = 0.0076 (GSK0660 1 µM)

Then, to determine the role of PPARβ/δ on MSC challenged by IR injury, cultured MSC were submitted to an IR-simulated stress in vitro using various concentrations of H_2_O_2_ (100, 250, 350, 500 and 750 µM) for 4 h (see the protocol on Fig. 1a). Apoptosis quantification was assessed by the mean of specific DNA fragmentation measurement for the different groups of treatment. We found that specific DNA fragmentation was induced by H_2_O_2_ as low as 100 µM with an equivalent rate for all concentrations from 100 to 750 µM (Fig. 1b). The concentration of 250 µM of H_2_O_2_ was chosen because it was the lowest dose providing the maximal effect compared to the control MSC (p****). To determine the role of PPARβ/δ on the resistance of MSC to H_2_O_2_, we pre-treated them with different concentrations (0.1 and 1 µM) of PPARβ/δ antagonist and agonist for 24 h before the H_2_O_2_-stress induction (250 µM for 4 h). MSC pre-treatment with the selective PPARβ/δ agonist GW0742 improved significantly the capacity of MSC to resist against H_2_O_2_-induced stress at 0.1 µM (p****) and 1 µM (p****). By contrast, the pre-treatment with the PPARβ/δ antagonist GSK0660 slightly protected the MSC against apoptosis at the concentration 0.1 µM (p*) but not at 1 µM (*p*^ns^ vs H_2_O_2_ group, Fig. 1c). The enhanced resistance to apoptosis induced by PPARβ/δ agonist pre-treatment on MSC was confirmed by a significant increase in B-cell lymphoma-2 (*bcl2)* gene expression level, in particular after a pre-treatment with GW0742 at 0.1 µM (p**). Conversely, the pre-treatment of MSC with 1 µM antagonist (GSK0660) significantly reduced *bcl2* gene expression level compared to H_2_O_2_-treated MSC (p**; Fig. 1d). In addition, we validated the effect of GW0742 PPARβ/δ agonist pre-treatment on cultured MSC and found that the agonistic effect of GW0742 was associated with increased expression levels of *Angiopoietin Like 4* (*angpl4)* target gene allowing to demonstrate the agonist functionality (Additional file 1: Fig. 2). Altogether, these results revealed that MSC activated for PPARβ/δ are protected from apoptosis induced by H_2_O_2_ stress.

### PPARβ/δ-priming increases the anti-apoptotic effect of MSC on cardiomyocytes exposed to an IR-simulated stress

**Fig. 2:**
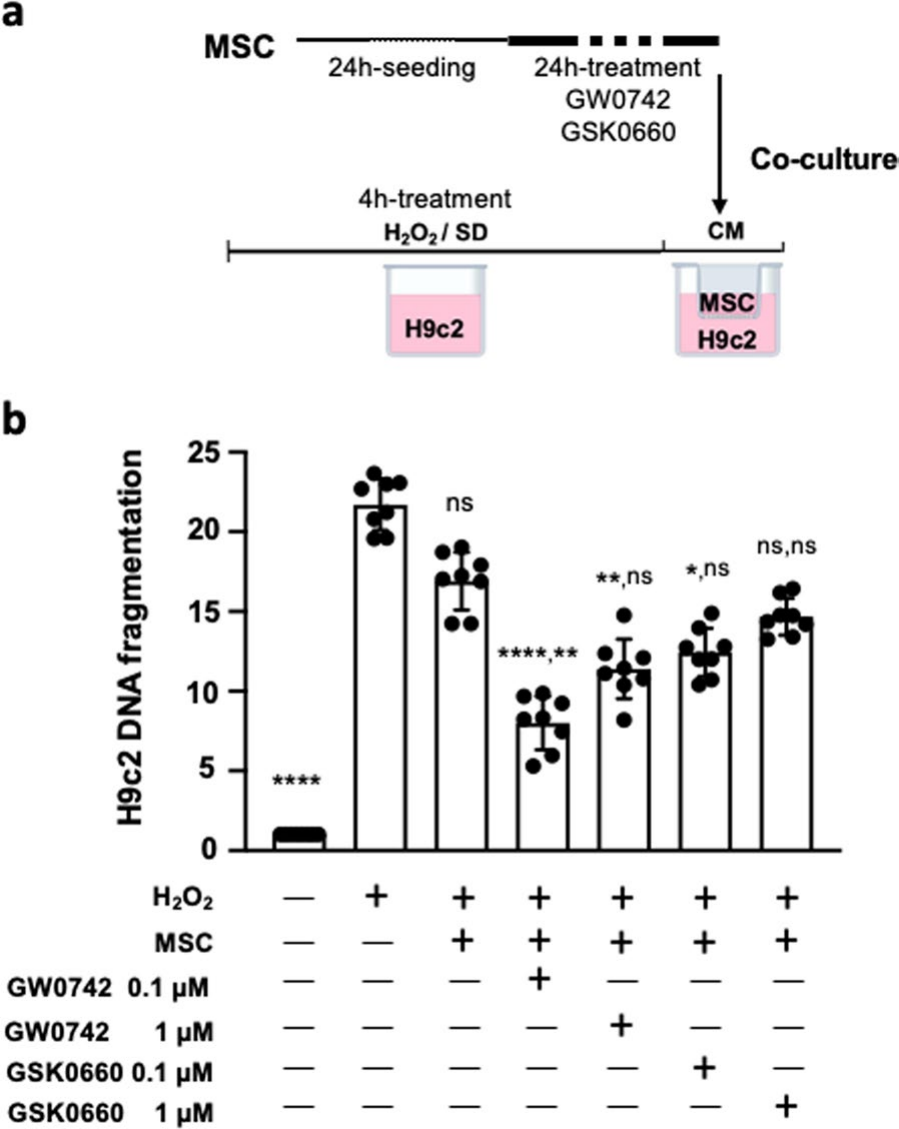
Increased anti-apoptotic effects of PPARβ/δ-primed MSC on cardiomyocytes. **a** After 24 h of MSC pharmacological preconditioning using PPARβ/δ agonist, cells were co-cultured in classical culture medium (CM) using transwells (upper chamber) with H9c2 (lower chamber) previously exposed to 350 µM of H_2_O_2_ in minimal medium (serum deprivation) during 4 h. At the end of the protocol, specific DNA fragmentation was quantified in H9c2 cells and values were compared with those obtained for co-cultures with naive MSC or MSC pre-treated with PPARβ/δ antagonist (GSK0660). **b** Scatter dot plot and bars were presented for specific DNA fragmentation quantified in H9c2 at the end of the protocols. Data (mean ± SD for each group of treatment) were normalized by values obtained with H9c2 (not challenged by H_2_O_2_) and compared among groups performed using Kruskal–Wallis with the Dunn’s post hoc test for multiple comparison. On the top of each bar, P values were noted for the comparison vs H9c2/H_2_O_2_ first and second vs MSC. P values vs H9c2/H_2_O_2_ were noted ns for *p* > 0.999 (naïve MSC), **** for *p* < 0.0001 (0.1 µM GW0742), vs ** for *p* = 0.073 (1 µM GW0742), * for *p* = 0.0402 (vs 0.1 µM GSK0660) and ns for *p* = 0.0582 (MSC/H_2_O_2_ vs 1 µM GSK0660). Significance compared to naïve MSC was noted ** for *p* = 0.0058 (0.1 µM GW0742), ns for *p* = 0.3642 (for 1 µM GW0742) and ns for *p* > 0.999 (for GSK0660 at both doses)

To determine the role of PPARβ/δ on the anti-apoptotic properties of MSC on stressed cardiomyocytes, we co-cultured in transwell systems naïve MSC and MSC activated or inhibited for PPARβ/δ with the H9c2 cardiomyoblast cell line, considered as a suitable model to study cardiac ischemia–reperfusion injury [47]. The protocol consisted on exposing H9c2 cardiomyoblasts to 350 µM of H_2_O_2_ for 4 h prior to their co-culture for 1 h with naive MSC or MSC modulated for PPARβ/δ (Fig. 2a). We showed that MSC pre-treatment with 0.1 µM of PPARβ/δ agonist GW0742 allowed to reveal a significant protective effect in H9c2 cells against H_2_O_2-_induced stress (MSC/0.1 µM GSK0742 vs untreated H9c2, *n* = 8 per group; p**** < 0.0001) that was not seen with naïve MSC (MSC vs untreated H9c2; *n* = 8 per group; *p*^ns^ = 0.999 and MSC/0.1 µM GW0742 vs MSC, *n* = 8 per group; p** = 0.0058; Fig. 2b). This anti-apoptotic effect was also observed in MSC pre-treated with 1 µM GW0742 (MSC/1 µM GW0742 vs untreated H9c2, *n* = 8 per group; p** = 0.0079). Pre-treatment of MSC with the GSK0660 did not change MSC protective properties compared to naïve MSC (MSC/0.1 µM and GSK0660 MSC/1 µM GSK0660 vs MSC, *n* = 8 per group; *p*^ns^ > 0.999). These results show that PPARβ/δ activation revealed and enhanced MSC anti-apoptotic properties on cardiomyocytes exposed to an IR-simulated stress in vitro.

### PPARβ/δ-priming increases the anti-apoptotic effect of MSC on endothelial cells exposed to an IR-simulated stress

**Fig. 3:**
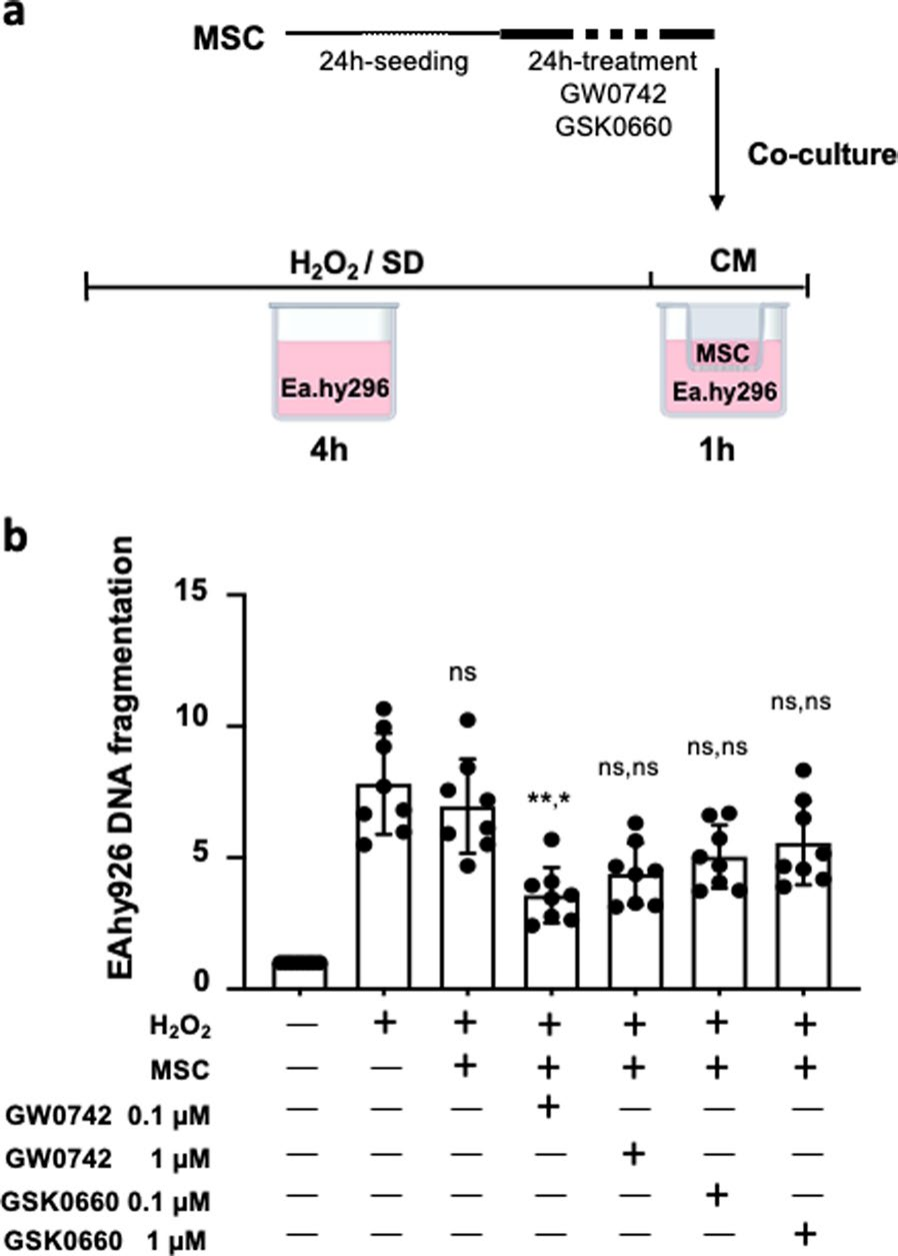
Increased anti-apoptotic effects of PPARβ/δ-primed MSC on endothelial cells. **a** After 24 h of MSC pharmacological preconditioning using PPARβ/δ agonist, cells were co-cultured in classical culture medium (CM) using transwells (upper chamber) with EA.hy296 (lower chamber) previously exposed to 350 µM of H_2_O_2_ in minimal medium (serum deprivation) during 4 h. At the end of the protocol, specific DNA fragmentation was quantified in EA.hy926 cells and values were compared with those obtained for co-cultures with naive MSC or MSC pre-treated with PPARβ/δ antagonists (GSK0660). **b** Scatter dot plot and bars were presented for specific DNA fragmentation quantified in EA/hy926 at the end of the protocols. Data (mean ± SD for each group of treatment) were normalized by values obtained with EA.hy926 (not challenged by H_2_O_2_) and compared among groups performed using Kruskal–Wallis with the Dunn’s post hoc test for multiple comparison. On the top of each bar, P values were noted for the comparison first vs EA.hy926/H_2_O_2_ and second vs MSC. P values vs EA.hy026/H_2_O_2_ were noted ns for *p* > 0.999 (naïve MSC), ** for *p* = 0.0061 (MSC/0.1 µM GW0742), ns for *p* = 0.0994 (MSC/1 µM GW0742), ns for *p* = 0.8650 (MSC/0.1 µM GSK0660) and ns for *p* > 0.999 (MSC/ 1 µM GSK0660). Significance compared to naïve MSC was noted * for *p* = 0.0347 (MSC/0.1 µM GW0742), ns for *p* = 0.3957 (for MSC/1 µM GW0742) and ns for *p* > 0.999 (MSC/GSK0660 at both doses)

Given the major importance of endothelial cells in the cardiac tissue repair process and their function, the enhancement of MSC anti-apoptotic properties on endothelial cells is critical. In order to evaluate the anti-apoptotic effect of PPARβ/δ-primed MSC, specific DNA fragmentation was quantified in EA.hy926 human endothelial cells stressed with H_2_O_2_ and co-cultured with MSC pre-treated or not with GW0742 (PPARβ/δ agonist). Figure 3 shows that MSC priming with 0.1 µM of GW0742 allowed to reveal the protective effect of MSC on H_2_O_2_-stressed endothelial cells (MSC vs MSC/GW0742 0.1 µM, p* = 0.0347). MSC priming with 1 µM GW0742 was not efficient enough to improve significantly MSC anti-apoptotic effects despite a great tendency (MSC vs MSC/GW0742 1 µM, *p*^ns^ = 0.3957). Indeed, MSC primed with 0.1 µM GW0742, by contrast to naïve MSC that were ineffective on their own (MSC vs untreated EA.hy926, *p* > 0.999), provided a significant protective effect on endothelial cells exposed to an H_2_O_2_-induced oxidative stress (untreated EA.hy926 vs MSC/GW0742 0.1 µM, p** = 0.0061). Of note, pre-treatment of MSC with the GSK0660 (PPARβ/δ antagonist) did not change MSC protective properties compared to naïve MSC (MSC/ GSK0660 0.1 µM and MSC/1 µM GSK0660 vs MSC, *n* = 8 per group; *p* > 0.999). These results show that PPARβ/δ activation unveils and potentiates MSC anti-apoptotic properties on endothelial cells exposed to an IR-simulated stress in vitro.

### *The cardioprotective effect of MSC administered during reperfusion in an *ex vivo* model of myocardial IR injury depends on PPARβ/δ*

**Fig. 4:**
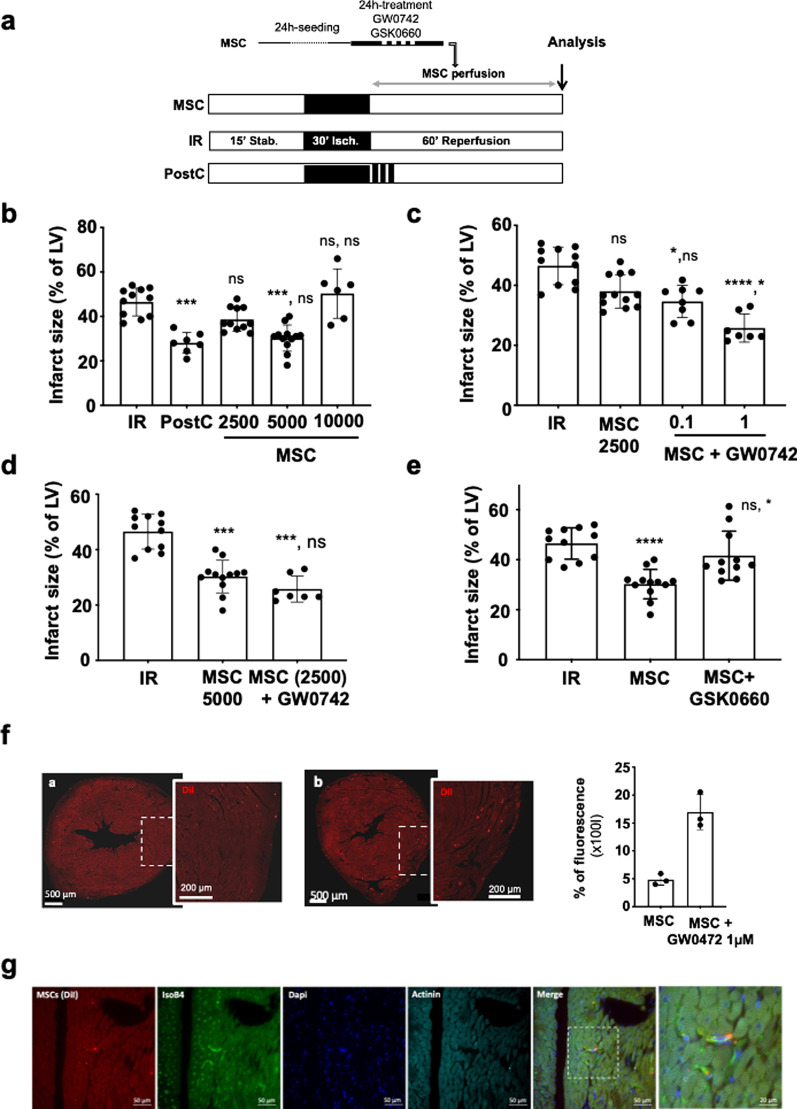
Increased cardioprotection in hearts treated by PPARβ/δ-primed MSC. **a** C57Bl6 mouse hearts were mounted on a Langendorff system and subjected to IR injury. The ex vivo protocol comprises a 15 min-period of stabilization, followed by 30 min of global ischemia achieved by stopping the flow through the aorta (no-flow). Reperfusion was achieved by restoring the Tyrode perfusion during 60 min (IR group). For the PostC group, a postconditioning stimulus comprising 3 cycles of 1 min ischemia-1 min reperfusion was applied at the onset of reperfusion. In the MSC group, reperfusion was achieved with a solution of MSC cells prepared in a Tyrode buffer at various concentrations (2500; 5000 or 10,000 cells/mL). At the end of the protocol, hearts were dedicated to infarct size (TTC-staining method; **b–e**) or immunochemistry (**f**) analysis. **b** Scatter plot and bars (mean ± SD) were represented for Infarct size in IR (*n* = 11), PostC (*n* = 7), MSC 2500 cells/mL (*n* = 11), MSC 5000 cells/mL (*n* = 10), and MSC 10,000 cells/mL (*n* = 10); Statistical analysis was performed using Kruskal–Wallis with the Dunn’s post hoc test for multiple comparison. Statistical significance compared to IR is noted *** for *p* = 0.0004 (PostC vs IR), ns for *p* = 0.8021 (MSC 2500 vs IR), *** for *p* = 0.0004 (MSC 5000 vs IR), ns for *p* > 0.99 (MSC 10,000 vs IR) and for comparisons vs PostC: ns for *p* = 0.097 (MSC 2500), ns for *p* > 0.9999 (MSC 5000), ** for *p* = 0.011 (MSC 10,000). **c** Scatter plot and bars (mean ± SD) were represented for Infarct size in IR (*n* = 11), MSC 2500 cells/mL (*n* = 12), MSC (2500 cells/mL) + GW0742 0.1 µM (*n* = 8), MSC (2500 cells/mL) + GW0742 1 µM (*n* = 7) and MSC (2500 cells/mL). Statistical analysis was performed by Kruskal–Wallis followed by the Dunn’s post-test. Statistical significance versus IR was noted ns for *p* = 0.0881 (MSC), * for *p* = 0.0309 (MSC/GW0742 0.1 µM) and **** for *p* < 0.0001 (MSC/GW0742 1 µM) and versus MSC: ns for *p* > 0.999 (MSC/GW0742 0.1 µM), * for *p* = 0.0429 (MSC/GW0742 1 µM). **d** Scatter plot and bars (mean ± SD) were represented for Infarct size in IR (*n* = 11), MSC 5000 cells/mL (*n* = 12) and MSC (2500 cells/mL) /1 µM GW0742 (*n* = 7). Statistical analysis was performed by Kruskal–Wallis followed by the Dunn’s post test. Statistical significance was noted *** for *p* = 0.001 (MSC 5000 vs IR), *** for *p* = 0.0003 (MSC 2500/GW0742 vs IR) and ns for *p* > 0.9999). **e** Scatter plot and bars (mean ± SD) were represented for Infarct size in IR (*n* = 11), MSC 5000 cells/mL (*n* = 12), MSC (5000 cells/mL) + GSK0660 (*n* = 11). Statistical analysis was performed by ANOVA (since the normality test passed) followed by the Tukey’s multiple comparisons test. Statistical significance was noted **** for *p* < 0.0001 (MSC vs IR), ns for *p* = 0.3467 (GSK0660 vs IR) and * for *p* = 0.0220 (MSC vs GSK0660). **f** Left panel: Representative pictures of microscopic observations for an MSC-treated heart section with corresponding enlarged immunostaining images (Original magnification: × 40 oil immersion) showing DI-I labelled **(a)** MSC and (**b**) MSC pre-treated with GW0742 1 µM in left ventricle collected 60 min after reperfusion (same time than for infarct size evaluation). Right panel: Scatter plots for the % of fluorescence measured in each slice of MSC versus MSC + GW0742 1 µM injected hearts (*n* = 3 for each group). **g** Representative pictures of microscopic observations of immunostaining from MSC-treated LV sections showing MSC (DiI, red labeling), vessels (IsoB4, green), cell nuclei (DAPI, blue) and cardiac actinin staining (anti-actinin antibody, cyan). The last picture represents the merge of the co-immunostaining showing that MSC are detected in microvessels at one hour of reperfusion. Original magnification: × 40 oil immersion; Scale bars are indicated on each picture

In order to evaluate the protective effect of MSC in an ex vivo model of myocardial IR injury, MSC were administered during the reperfusion phase at various concentrations (2500; 5000 and 10,000 cells/mL in a Tyrode solution) in isolated hearts subjected to global ischemia (see protocol, Fig. 4a). MSC showed a U-shaped dose–response curve with an optimal efficacy at 5000 cells/mL which was similar to that provided by the PostC (ischemic postconditioning) stimulus considered as the positive control in our experiment (30.27 ± 5.89, for MSC 5000, *n* = 12 vs 46.51 ± 6.28, *n* = 11 for IR; p*** = 0.0004 and vs 28.13 ± 4.69, *n* = 7 for PostC; p ^ns^ > 0.999) (Fig. 4b). In addition, neither the 2500 cells/mL dose (38.64 ± 5.36, *n* = 11 vs 46.51 ± 6.28, *n* = 11 for IR; *p*^ns^ = 0.80) nor the largest one tested, 10,000 cells/mL (50.28 ± 11.09, *n* = 6 for MSC 10,000 vs 46.51 ± 6.28, *n* = 11 for IR; *p*^ns^ > 0.999) produced a significant decrease in infarct size compared to IR.

Since, we demonstrated in previous studies the pivotal role of PPARβ/δ expression on MSC functions including their immunoregulatory properties, we addressed here its role on the cardioprotective potential of MSC [48–50]. To that end, MSC were pre-treated with either PPARβ/δ agonist or antagonist prior to their administration in the ex vivo model of IR injury. According to this result, we investigated whether the use of GW0742 PPARβ/δ agonist at various doses (0.1 and 1 µM) was associated with an improvement of the cardioprotection afforded by MSC administered at the lower dose (2500 cells/mL) that was not protective by itself (Fig. 4c). We showed that pre-treating MSC with 0.1 and 1 µM PPARβ/δ agonist induced their cardioprotective effect (46.51 ± 6.28, *n* = 11 for IR vs 34.65 ± 5.36, *n* = 8 for MSC/0.1 µM GW0742; p* = 0.0309 and vs 25.77 ± 4.68, *n* = 7 for MSC/1 µM GW0742; p**** < 0.0001). Pre-treating the cells with GW0472 (1 µM) before administration at 2500 cells/mL allowed to induce the same cardioprotective effect as the one observed using 5000 cells/mL naïve MSC (*p*^ns^ > 0.999; Fig. 4d). Figure 4e shows that the cardioprotection was abolished when MSC (5000 cells/mL) were pre-treated during 24 h with GSK0660 (1 µM) prior to their administration during the reperfusion phase (40.22 ± 7.24, *n* = 11 for MSC/GSK0660 vs 46.51 ± 6.28, *n* = 11 for IR, *p*^ns^ = 0.35467 and vs 30.27 ± 5.89, *n* = 12 for MSC; p* = 0.022).

This improvement of the therapeutic effect of PPARβ/δ-primed MSC as compared to their naïve counterpart was associated with an increased number of MSC in the microvessels in the infarcted myocardial tissue as assessed by immunohistology experiments using DiI-labeled MSC (Fig. 4f).

### *PPARβ/δ-priming increases the cardioprotective effects of MSC *ex vivo* by decreasing caspase 3 activation in IR heart*

**Fig. 5:**
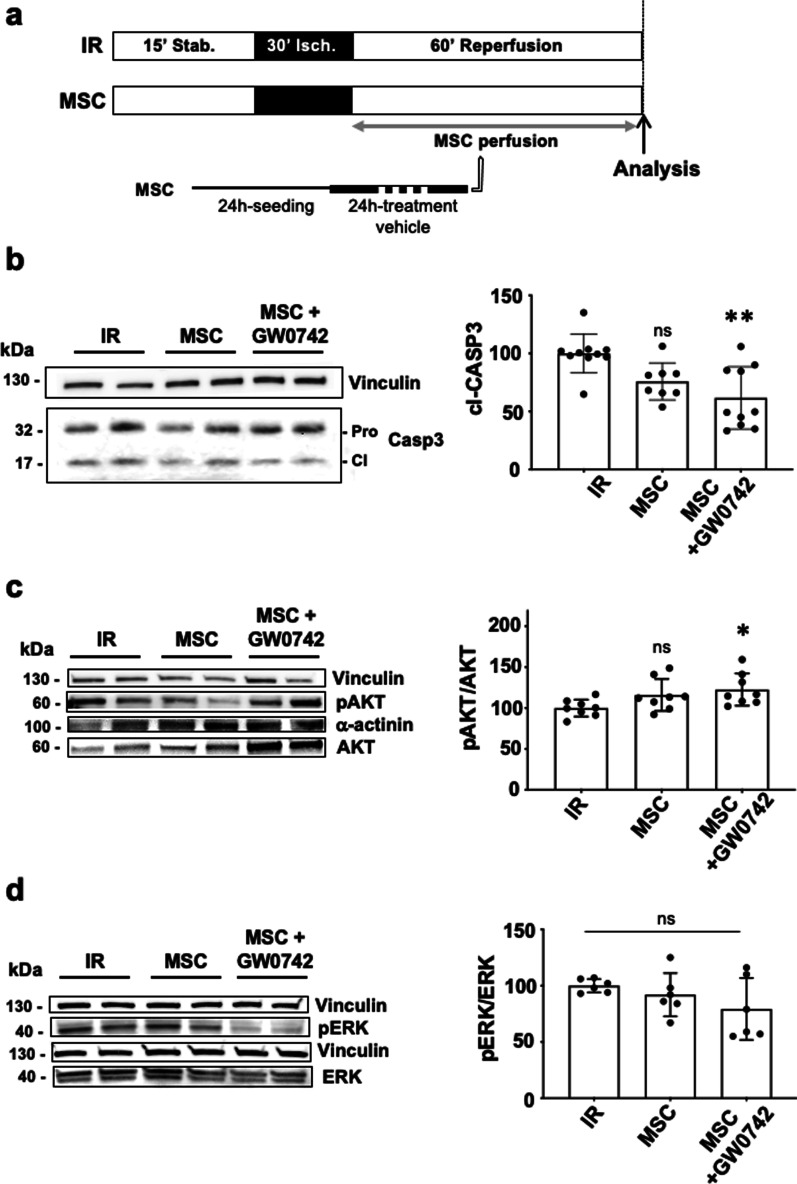
**a** C57Bl6 mouse hearts were mounted on a Langendorff system. The ex vivo protocol comprises a 15 min-period of stabilization, followed by 30 min of global ischemia achieved by stopping the flow through the aorta (no-flow). Reperfusion was achieved by restoring the Tyrode perfusion during 60 min (IR group). In the MSC group, reperfusion was achieved with a solution of MSC cells prepared in a Tyrode buffer (2500 cells/mL) pre-treated or not by GW0742 at 1 µM. At the end of the protocol, hearts were collected and proteins extracted for further analysis. **b, c, d** *Western blot* analysis was performed from LV protein extracts from IR non-treated or MSC-treated murine IR hearts (ex vivo) with or without a pharmacological pre-treatment. Scatter dot blots and mean ± SD were plotted for Cleaved-caspase 3 (p** = 0.064 for IR, *n* = 10 vs MSC/GW0742, *n* = 10), for pAKT/AKT (p* = 0.0385 for IR, *n* = 8 vs MSC/GW0742, *n* = 8) and for pERK/ERK (*p*^ns^ = 0.2476 for IR, *n* = 6 vs MSC/GW0742, *n* = 6). Representative gel blots are presented for each protein for the three conditions (IR, MSC and MSC + GW0742). Vinculin or α-actinin were used as protein loading control. Data were compared using non parametric Kruskal–Wallis test (Dunn’s post hoc test)

The potent anti-apoptotic effect of the PPARβ/δ-primed MSC on cardiomyocytes was further confirmed on whole hearts. Western blot analysis in hearts subjected ex vivo to IR injury allowed to show that pre-treating MSC during 24 h with the GW0742 PPARβ/δ agonist (1 µM; see protocol in Fig. 5a) allowed to reveal a significant inhibition of caspase 3 cleavage compared to the IR condition (p* = 0.0303 versus IR) that was not observed with naïve MSC (*p*^ns^ = 0.4986 vs IR; Fig. 5b). By contrast, the administration of MSC inhibited for PPARβ/δ in the ex vivo IR heart at reperfusion, did not reduce caspase 3 activation as compared to the IR control condition (data not shown).

In addition, evaluation of the phosphorylation patterns of AKT (proteine kinase B) and ERK1/2 (Extracellular signal-regulated kinase) pro-survival kinases showed a significantly increased phosphorylation pAkt/Akt ratio in MSC-treated versus non-treated IR hearts (Fig. 5c, d) when MSC were pre-treated with GW0742 PPARβ/δ agonist (1 µM) prior to their administration during reperfusion. By contrast, there was no change in the pERK1/2 /ERK1/2 ratio when naive MSC were administered in the post-ischemic phase of the IR protocol.

## Discussion

Our study is the first demonstration that the priming of murine MSC using an agonist of PPARβ/δ allows to protect them from apoptosis induced by an H_2_O_2_-induced stress and to enhance their anti-apoptotic and cardioprotective properties against myocardial IR injury ex vivo. First, we evidenced in vitro that PPARβ/δ priming allowed to increase MSC resistance against H_2_O_2_-induced oxidative stress as assessed by decreased apoptosis rate and by an increased amount of *bcl2* transcripts. Then, we observed that PPARβ/δ-primed MSC had a more potent anti-apoptotic effect on both cardiomyocytes and endothelial cells than naïve MSC. Interestingly, the priming of MSC with a PPARβ/δ agonist before their injection in an ex vivo model of myocardial IR injury allowed them to confer significant cardioprotective properties not observed with naïve MSC when evaluated in the same model. Importantly, PPARβ/δ-priming reduced by two the number of MSC administered to obtain the optimal efficiency in decreasing infarct size. This enhanced short-term cardioprotection, within 1 h after reperfusion, was also associated with an increased number of MSC detected in the ventricular wall and also enhanced anti-apoptotic effects as assessed by *western blot* analysis of caspase 3 activation. Accordingly, the phosphorylation ratio of AKT pro-survival kinase was increased in IR hearts treated by PPARβ/δ-primed versus naive MSC. Of note, the cardioprotective effects of MSC were lost when pre-treated with a PPARβ/δ antagonist prior to their administration, confirming our previous results using MSC derived from PPARβ/δ knockout mice.

Reperfusion, even if considered as a double-edged sword, is the only treatment recommended for patients with acute myocardial infarction [51]. Despite obvious beneficial effects in limiting the ischemic insult, revascularization of the culprit artery leads to an abrupt return of oxygen in the threatened myocardial tissue and generates subsequent deleterious side effects. Lethal reperfusion injury corresponds to the death of cardiac cells viable at the end of the ischemic period occurring at the onset of reperfusion and mainly related to apoptotic death [52, 53]. Regarding MSC-based therapy against myocardial IR injury, MSC undergo apoptosis in particular when they are transplanted in vivo in injured tissues, such as damaged hearts after IR injury [54]. Several strategies were developed to inhibit apoptosis of MSC after in vivo administration to help them survive hypoxia-reoxygenation injury, such as heat-shock treatment [55] or a hypoxia-regulated Heme oxygenase-1 (HO-1) vector modification [56] of MSC. Among the factors impacting negatively the therapeutic effect of MSC, the poor survival rate of transplanted cells is of major importance. So, MSC resistance to oxidative stress should be increased to improve their therapeutic efficacy against IR injury [57]. Here, we demonstrate for the first time that PPARβ/δ-priming of MSC enhanced their capacity to be protected against apoptosis when they were subjected to an H_2_O_2_-induced stress revealing that PPARβ/δ-priming is able to increase MSC resistance to oxidative stress in vitro. These results are in line with the anti-apoptotic effects of PPARβ/δ stimulation described, in vitro, on several cell types including endothelial cells, H9C2 cardiomyoblasts, neurons or keratinocytes [41–43]. In endothelial cells, PPARβ/δ anti-apoptotic properties were associated to an increased protection against oxidative stress [41, 42]. In the present study, RT-qPCR experiments have shown that MSC-increased resistance to H_2_O_2_-induced stress upon PPARβ/δ-priming *i.e.,* stimulation of the receptor by an agonist, was associated with an increased amount of anti-apoptotic *bcl2* gene transcripts. Other anti-apoptotic mechanisms have also been highlighted in other cell types following the activation of PPARβ/δ receptors, such as the activation of AKT1 pathway. Gamdzyk's team has shown in the context of neuronal stress that the activation of PPARβ/δ by its agonist GW0742 leads to the inhibition of the pro-apoptotic pathway ASK1/p38 MAPK [58]. In the context of PPARβ/δ MSC priming, the decreased rates of apoptosis after an H_2_O_2_-induced stress could be of major importance to explain the increase in their therapeutic properties. Indeed, we demonstrate for the first time that PPARβ/δ-priming of MSC was associated with their enhanced anti-apoptotic potential both in vitro and ex vivo. H9C2, described as a suitable model to study cardiac ischemia–reperfusion injury in vitro [47], were subjected to an H_2_O_2_-induced stressed prior to be co-cultured with MSC. Then, specific DNA fragmentation was measured as a hallmark of apoptosis in H9C2 co-cultured with PPARβ/δ-primed or naïve MSC. While approaches to improve MSC-based anti-apoptotic effects on cardiomyocytes have shown promising results both in vitro and in vivo [30, 32, 33, 59], very few studies have investigated the anti-apoptotic paracrine effects mediated by MSC on endothelial cells. Only data on tunneling nanotubes [60] and extracellular vesicles derived from MSC have been reported to repress high glucose (HG) [61] or serum deprivation-induced apoptosis [62, 63]. Our present results obtained on a human endothelial cell line reveal, for the first time, that MSC pharmacologically preconditioned are able to provide an enhanced paracrine anti-apoptotic effect. Ex vivo, a strong pro-apoptotic stress was assessed by *western blot* analysis of cleaved caspase 3 expression in isolated hearts subjected to severe IR injury showing a 46.5%-mean infarct size (expressed as a % of the LV, SHAM, *n* = 6 vs IR, *n* = 10; p** = 0.0047; data not shown). MSC-induced cardioprotection was associated with a decreased amount of cleaved (activated) caspase 3 measured in the left ventricle as already reported for MSC injected in the injured myocardium [64]. MSC priming with PPARβ/δ agonist exacerbated the anti-apoptotic properties, allowing in particular to unmask potent anti-apoptotic effects that were absent when using naïve MSC at the same dose. Targeting regulated cell death pathways, in particular apoptosis, after an ischemic event, appears as a main strategy to prevent reperfusion injury and to limit infarct size [7–16]. Anti-apoptotic strategies allow to inhibit specifically reperfusion-induced apoptotic burst of cell death, providing long-term cardioprotective effects, a strategy evidenced recently by our group in the mouse heart [65]. Moreover, considering vascular cells as a major target for cardioprotection is an important concept that could account for the failure in translation reported for cardioprotective studies against IR injury in which the role of the coronary circulation (in the myocardium) has been neglected up to now [66, 67].

In terms of cardioprotection, MSC administered during the reperfusion phase provided a 35%-decrease in infarct size. Regarding their concentration, we observed a U-shaped curve clearly showing that MSC-induced cardioprotection is dose-dependent as reported in clinical trials. Indeed, in AMI patients, a recent study reported that only doses less than 10^7^ MSC administered within 1 week after AMI enhanced the systolic function of left ventricle whereas administration of more than 10^7^ cells had the opposite effect [68]. In agreement with this study, a U-shaped curve for MSC cardioprotective effects in the treatment of chronic advanced ischemic heart failure was also reported indicating reduced outcomes at the highest dose of cells [69]. The U-shape curve in our study allowed to identify the optimal dose providing a maximal cardioprotective effect (5000 cells/mL administered during 60 min of reperfusion). The corresponding number of cells that was administered *per* heart was around 7.5 × 10^5^ cells, which corresponds to a relatively low dose compared to other reports in the literature. The use of a higher dose of MSC induced the loss of cardioprotection. Interestingly, we observed also that MSC primed with a PPARβ/δ agonist allowed to decrease the dose of MSC injected in the heart by half. Indeed, in our study, the cardioprotective effect observed ex vivo using PPARβ/δ-primed MSC injected at a dose of 3.75 × 10^5^ MSC was similar to that obtained with naive MSC administered at the optimal dose (7.5 × 10^5^ MSC).

Inhibition of PPARβ/δ activity prior to their administration ex vivo in IR injured hearts provided the same results obtained in our previous study using MSC derived from PPARβ/δ knockout mice that failed to protect myocardial tissue against IR injury ex vivo [70]. By contrast, MSC priming with GW0742 PPARβ/δ agonist allowed to unmask and potentiate their cardioprotective effects. For the optimal dose of GW0742 agonist used to prime MSC, a shift from 0.1 to 1 µM was observed between in vitro and ex vivo results. It seems reasonable to speculate that this difference may be due to the fact, that the ex vivo system integrates several cell types including fibroblast, endothelial but also resident immune cells and that effect of MSC might differ depending on the cell type with which they interact.

## Conclusions

Our study provides evidence for an enhanced MSC-based cell product inducing cardioprotection against IR injury with potent anti-apoptotic effects and an increased number of surviving MSC in the injured myocardium, one hour after the onset of reperfusion.

These results could be of major interest in the clinical setting to improve MSC efficacy for the cardioprotection of injured myocardium in AMI patients.

## Supplementary Information

**Additional file 1**. Supplementary figures.

## Data Availability

The datasets during and/or analyzed during the current study available from the corresponding author on reasonable request.

## Abbreviations

AKT: Protein kinase B
ALP: Alkaline phosphatase
AMI: Acute myocardial infarction
angpl4: Angiopoietin like 4
bcl2: B-cell lymphoma-2
bFGF: Basic fibroblast growth factor
BCA: Bicinchoninic acid
Col2: Collagen type II
cl-CASP3: Cleaved caspase 3
CM: Culture medium
DMEM: Dulbecco’s modified Eagle’s medium
ERK1/2: Extracellular signal-regulated kinase
EV: Extracellular vesicles
FBS: Fetal bovine serum
HO1: Heme oxygenase-1
IR: Ischemia–reperfusion
MSC: Mesenchymal Stromal Cells
MEM: Minimum essential médium
min: Minutes
OC: Osteocalcin
PPAR: Peroxisome proliferator-activated receptors
PBS: Phosphate Buffer Saline
PostC: Postconditioning
RXR: Retinoid X receptor
RT-qPCR: Reverse Transcriptase-quantitative Polymerase Chain Reaction
RPS9: Ribosomal protein S9
