# Correction: PPARβ/δ priming enhances the anti-apoptotic and therapeutic properties of mesenchymal stromal cells in myocardial ischemia–reperfusion injury

**DOI:** 10.1186/s13287-022-03086-6

**Published:** 2022-07-26

**Authors:** Charlotte Sarre, Rafael Contreras-Lopez, Nitirut Nernpermpisooth, Christian Barrere, Sarah Bahraoui, Claudia Terraza, Gautier Tejedor, Anne Vincent, Patricia Luz-Crawford, Kantapich Kongpol, Sarawut Kumphune, Christophe Piot, Joel Nargeot, Christian Jorgensen, Farida Djouad, Stéphanie Barrere-Lemaire

**Affiliations:** 1 IGF, Université de Montpellier, CNRS, INSERM, 141 rue de la Cardonille, 34094 Montpellier Cedex 5, France; 2 IRMB, Univ Montpellier, INSERM, Montpellier, France; 3 IBRU, Department of Cardio-Thoracic Technology, Faculty of Allied Health Sciences, Naresuan University, Phitsanulok, Thailand; 4 Laboratorio de Inmunología Celular Y Molecular, Facultad de Medicina, Universidad de los Andes, Santiago, Chile; 5 IMPACT, Center of Interventional Medicine for Precision and Advanced Cellular Therapy, Santiago, Chile; 6 School of Allied Health Sciences, Walailak University, Nakhon Si Thammarat, Thailand; 7 Département de Cardiologie Interventionnelle, Clinique du Millénaire, Montpellier, France; 8 CHU Montpellier, 34295 Montpellier, France; 9 Biomedical Engineering Institute, Chiang Mai University, Chiang Mai, Thailand

## Correction to: Stem Cell Research & Therapy (2022) 13:167 10.1186/s13287-022-02840-0

The original article contained errors in the attribution of affiliations which have since been corrected.

