# Supplementary material for: PPARβ/δ priming enhances the anti-apoptotic and therapeutic properties of mesenchymal stromal cells in myocardial ischemia–reperfusion injury
Source: Stem Cell Res Ther. 2022 Apr 23;13:167. doi: 10.1186/s13287-022-02840-0 (PMC9034535; doi:10.1186/s13287-022-02840-0)
Supplement: Supplementary file 1 — Additional file 1. Supplementary figures. [file 13287_2022_2840_MOESM1_ESM.pdf]

Additional File 1

PPARβ/δ priming enhances the anti-apoptotic and therapeutic properties of mesenchymal stromal cells in myocardial ischemia-reperfusion injury

By

Charlotte Sarre[1,2$], Rafael Contreras-Lopez[1,2$], Nitirut Nerpernpisooth[3], Christian Barrere[1], Sarah Bahraoui[2], Claudia Terraza[2], Gautier Tejedor[2], Anne Vincent[1], Patricia Luz-Crawford[4,5], Kantapich Kongpol[1,3], Sarawut Kumphune[6], Christophe Piot[1,7], Joel Nargeot[1], Christian Jorgensen[2,8], Farida Djouad[2,#], Stéphanie Barrere-Lemaire[1,#]

[$] Equally contributing first authors; [#] Equally contributing corresponding authors

Supplementary figures

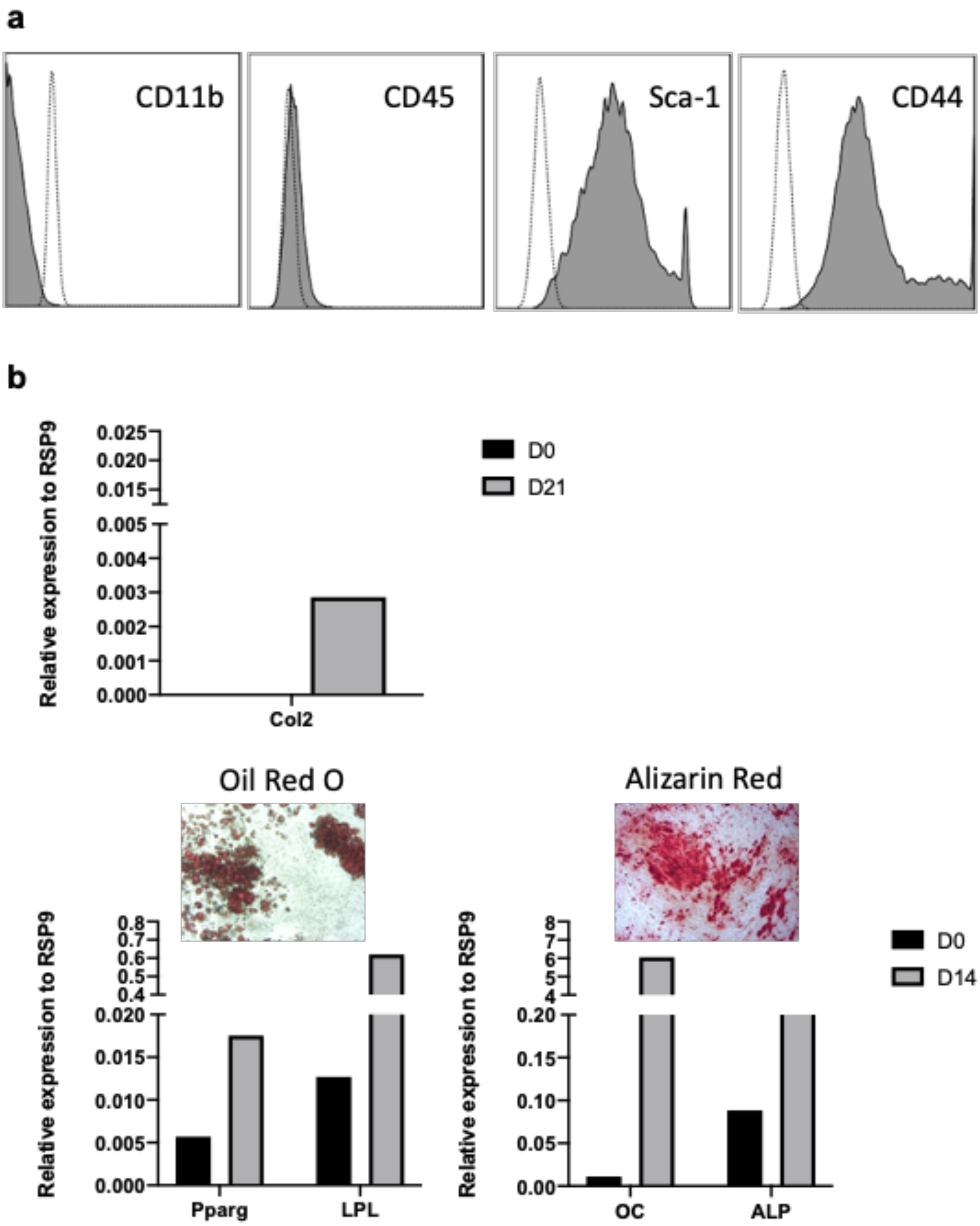

<u>Supplementary Figure 1.</u> **Characterization of murine MSC derived from the bone marrow of C57BL/6 mice. a** Phenotype of MSCs was assessed by flow cytometry. Differentiation potential of MSC. **b** The chondrogenic differentiation of MSC was assessed by

RT-qPCR to measure the relative expression of *collagen type II* (*Col2*) a marker characteristic of chondrocytes. The adipogenic potential of MSC was evaluated by RT-qPCR to measure the relative expression of *lpl* and *pparγ*, markers characteristic of adipocytes, and by the visualization of lipid droplets by phase contrast microscopy at day 14 of the differentiation process. Finally, the osteogenic differentiation potential of MSC was evaluated by RT-qPCR to measure the relative expression of *osteocalcin* (*oc*) and *alkaline phosphatase* (*alp*), markers characteristic of osteoblasts, and by Alizarin Red S positive staining.

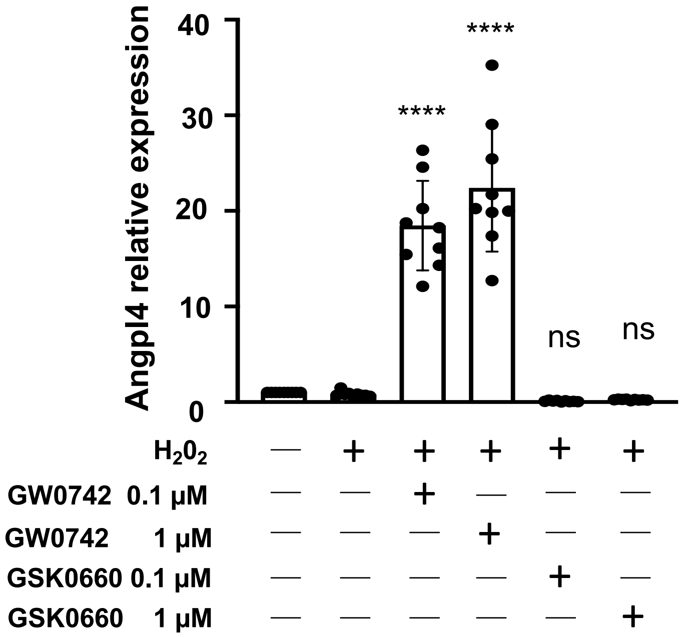

Supplementary Figure 2:

Agonist functionality of GW0742 on PPARβ/δ was assessed by RT-qPCR to measure the relative expression of the *Angpl4 target gene*. mRNA expression levels in untreated or preconditioned MSC exposed to H_2O_2 (250 µM) were assessed by RT-qPCR. Data (mean ± SD; n=9 for each group) normalized to the control values (data obtained for MSC without H_2O_2)

were compared using ANOVA and the Tukey's post hoc test (normality test passed). P values *vs* MSC/H_2O_2 were noted in **(A):** **** for $p<0.0001$ and ns for $p>0.999$ and in (**B**): *** for $p=0.0001$, ** for $p=0.0046$, * for $p=0.0140$ and ns for $p=0.1006$.
